# Effects of different drugs and hormone treatment on *Toxoplasma gondii* glutathione S-transferase 2

**DOI:** 10.1186/s13071-022-05589-w

**Published:** 2022-12-12

**Authors:** Shuang Li, Zhu Ying, Yangfei Xue, Zhepeng Sun, Jing Liu, Qun Liu

**Affiliations:** 1 Key Laboratory of Animal Epidemiology of Ministry of Agriculture, National Animal Protozoa Laboratory, College of Veterinary Medicine, China Agricultural University, Yuanmingyuan West Road, Haidian District, Beijing, China; 2 Department of Parasitology, Medical College, Zhengzhou University, Zhengzhou, 450052 China

**Keywords:** *Toxoplasma gondii*, Glutathione S-transferase, Progesterone, Drugs

## Abstract

**Background:**

Glutathione S-transferase (GST) in eukaryotic organisms has multiple functions such as detoxifying endogenous/exogenous harmful substances to protect cells from oxidative damage, participating in sterol synthesis and metabolism, and regulating signaling pathways. Our previous work identified an important GST protein in *Toxoplasma* that contributes to vesicle trafficking called TgGST2, the deletion of which significantly reduces the virulence of the parasite. Meanwhile, we considered that TgGST2 may also play a role in other pathways of parasite life activities.

**Methods:**

The tertiary structures of TgGST2 as well as estradiol (E2) and progesterone (P4) were predicted by trRosetta and Autodock Vina software, the binding sites were analyzed by PyMol's GetBox Plugin, and the binding capacity was evaluated using Discovery Studio plots software. We examined the influence of E2 and P4 on TgGST2 via glutathione S-transferase enzyme activity and indirect immunofluorescence assay (IFA) and through the localization observation of TgGST2 to evaluate its response ability in different drugs.

**Results:**

TgGST2 could bind to exogenous E2 and P4, and that enzymatic activity was inhibited by the hormones in a concentration-dependent manner. Upon P4 treatment, the localization of TgGST2 changed from Golgi and vesicles to hollow circles, leading to abnormal localization of the molecular transporter Sortilin (VPS10) and microneme proteins (M2AP and MIC2), which ultimately affect the parasite life activities, but E2 had no significant effect. Moreover, diverse types of drugs had divergent effects on TgGST2, among which treatment with antifungal agents (voriconazole and clarithromycin), anticarcinogens (KU-60019, WYE-132 and SH5-07) and coccidiostats (dinitolmide and diclazuril) made the localization of TgGST2 appear in different forms, including dots, circles and rod shaped.

**Conclusions:**

Our study shows that TgGST2 plays a role in sterol treatment and can be affected by P4, which leads to deficient parasite motility. TgGST2 exerts divergent effects in response to the different properties of the drugs themselves. Its responsiveness to diverse drugs implies a viable target for the development of drugs directed against *Toxoplasma* and related pathogenic parasites.

**Graphical Abstract:**

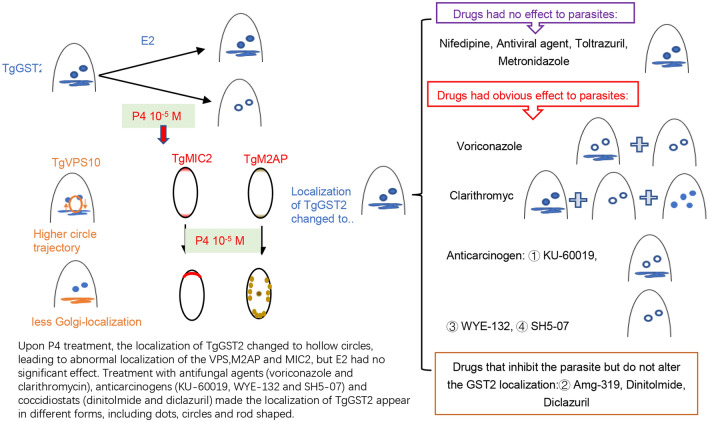

**Supplementary Information:**

The online version contains supplementary material available at 10.1186/s13071-022-05589-w.

## Background

*Toxoplasma gondii* is an important zoonotic parasite belonging to the apicomplexan phylum that infects immunocompromised humans and many other animals, causing severe diseases in the host [1]. The damage and clinical manifestations of *T. gondii* on the host depend mainly on its virulence and the species, age and immunity of the host. Infection rates are high in both humans and animals, but only under some special physiological or pathological conditions can *T. gondii* lead to toxoplasmosis and complications of varying severity. Once invaded into cells, *T. gondii* begins to form a parasitophorous vacuole (PV) for proliferation inside, leading to acute infection [2]. Tachyzoites undergo transformation into bradyzoites in response to host immune stress and persist as latent tissue cysts for long-term existence, especially in the brain, nerves and muscles, which transmit the parasite to other hosts via predation [3, 4].

Liesenfeld et al. had demonstrated that female animals infected with *Toxoplasma* were more likely to die compared to males because of a greater number of tachyzoites in the small intestine of females. Treatment of female mice with testosterone reduces the number and pathology of intestinal parasites. Thus, sex hormones are important factors in the susceptibility to *T. gondii* infection [5]. Thus far, our team has explored the effects and mechanism of estradiol (E2) and progesterone (P4) on *Toxoplasma* infection. Estradiol could promote the invasion of Pru and VEG strains into various host cells and enhance the pathogenicity of *T. gondii* in mice. Microneme protein 2 (MIC2) is the most representative key protein mediating the motility of the parasite [6]. Zhang et al. revealed that estradiol can enter the cytoplasm of parasites, rapidly induce intracellular calcium flux through PI-PLC and PKG pathways, promote the secretion of Tg-MIC2 and accelerate the gliding and egress ability of parasites, thus promoting parasite activity and pathogenicity in mice [7]. As to progesterone, which failed to promote the invasion of *T. gondii*, it inhibited the growth of parasites and their pathogenicity in mice because of the reduced secretion of TgMIC2 and reduced gliding ability. Meanwhile, parasites cultured with progesterone resulted in abnormal division and disordered formation of the inner membrane complex [8].

GSTs in mammalian cells are always involved in steroid biosynthesis and metabolism, especially sexual steroids, affecting their transport and regulating gene transcription and expression [9]. In vitro, human alpha-class glutathione transferase 3–3 (hGSTA3-3) catalyzes steroid Δ^5^–Δ^4^ double-bond isomerization using glutathione as a cofactor [10]. It was also involved in the biosynthesis of steroid hormones in complex cellular systems, thus helping 3β-hydroxysteroid II dehydrogenase (HSD) to catalyze the metabolism of Δ^5^-precursors into dehydroepiandrosterone and progesterone, and the catalytic efficiency of αGST in sterol-producing tissues was 230- and 25-fold higher than that of 3β-HSD alone [9]. Estrogen receptor α (ERα) plays an important role in the estrogen-mediated signaling pathway, binding to estrogen response elements (ERE) to perform multiple functions, and its abnormal regulation always leads to multiple diseases. Human GSTP1-1 could inhibit the expression of estrogen receptor α (ERα)-mediated negative regulatory receptor interaction protein 140 (RIP140) at the mRNA and protein levels and is an important regulator of the estrogen receptor pathway [11].

Apart from the above functions, GSTs are also a family of detoxification enzymes that catalyze the conjugation of GSH to various kinds of endogenous and exogenous compounds [12]. Exogenous substrates of soluble GST include drugs, industrial intermediates, pesticides, herbicides, environmental pollutants, etc. [13]. The effectiveness of GST against alien substances depends on the combined action of glutamate cysteine ligase and glutathione synthase. On the one hand, glutamate cysteine ligase and glutathione synthase provide a continuous supply of GSH, which binds to heterologous substances. On the other, transporters such as multidrug resistance-associated protein (MRP) contribute to the removal of the external glutathione conjugate [14]. GST can also catalyze the binding of GSH to electrophilic groups of endogenous products produced by oxidative damage for detoxification, such as membrane lipid peroxides and oxidative DNA degradation products [15, 16]. It is also an important regulator of cell proliferation and death signal transduction pathways [17] and is involved in the growth and differentiation of cancer cells and resistance to anticancer drugs, making it the most attractive drug target [18, 19].

Our previous work on *Toxoplasma* GSTs had demonstrated that TgGST2, located in Golgi (short rod-like localization prior to the nuclei) and some vesicles (solid dot-like localization in the endosome system), has a function similar to that of plant GST—targeted transmembrane transport—and is an important factor affecting parasite proliferation and virulence to mice [20]. Protein hormones produced by neuroendocrine cells are stored in vesicles in large amounts and are released via exocytosis when stimulated so that the body can quickly access them when needed. For example, the concentration of prolactin (PRL) in secretory granules is 200 times higher than in the endoplasmic reticulum lumen [21]. Considering the vesicular localization of TgGST2, we wondered whether it also plays a role in other aspects, such as involvement in sterol metabolism and handling of toxic compounds, thus influencing the life activity of *T. gondii*.

## Materials and methods

### Host cells and toxoplasma culture

Gene editing parasites (TgGST2-HA, *∆gst2* and FLAG-tagged Rab5/Rab6/Rab7/VPS10) were previously constructed [20]. Parasites were continuously cultured in African green monkey kidney (Vero) cells and human foreskin fibroblast (HFF, maintained in our laboratory) cells using Dulbecco’s modified Eagle’s medium (DMEM) supplemented with 2% fetal bovine serum (FBS) at 37 °C and 5% CO_2_.

### Glutathione S-transferase enzyme activity

The detection of GST enzyme activity was carried out using 1-chloro-2, 4-dinitrobenzene (CDNB) as a substrate according to previous descriptions [20, 22]. Estradiol and progesterone were dissolved in DMSO and diluted at different concentrations, before being mixed separately with rTgGST2 and incubated at 37℃ for 1 h. Reduced glutathione (GSH, 20 mM) was added to the above mixture and mixed with different concentrations of CDNB in Tris–HCl (100 mM, pH = 7.5) buffer in a 96-well plate, and their changes in absorbance values were measured by BioTek Epoch at OD340 within 5 min at room temperature.

### Construction of a tertiary structure model of TgGST2 and virtual docking with progesterone and estradiol

The amino acid sequence of TgGST2 was entered into the protein tertiary structure prediction online website trRosetta (https://yanglab.nankai.edu.cn/trRosetta/), and the highest scoring prediction model was selected to assess its quality. Aided by the tertiary structure model, the RaptorX—Binding Site (http://raptorx.uchicago.edu/BindingSite/) was used to predict the active site for binding to small molecules, and its three-dimensional structure was determined by the PyMol's GetBox Plugin software.

After preprocessing the tertiary structure of TgGST2 as well as progesterone and estradiol by AutoDock Vina software, TgGST2 was molecularly docked to progesterone and estradiol, respectively, using a semi-flexible docking method. The lowest binding energy was selected to map the molecular interactions of the tertiary structure by PyMol software, and the molecular interactions of the secondary structure were predicted using Discovery Studio plots software.

### Indirect immunofluorescence assay

HFFs grown on glass coverslips were infected with parasites and incubated in a CO_2_ incubator at 37 ℃ for 20 h, followed by fixation with 4% paraformaldehyde for 15 min. Cells were permeabilized with 0.25% Triton-X 100 for 20 min and blocked with 3% BSA for 30 min. Cells were incubated with primary antibodies for 1 h, washed three times and then incubated with secondary antibody for 1 h. Nuclei were stained with Hoechst at 1:100 dilution. Mouse anti-HA and mouse anti-FLAG antibodies were purchased from Sigma; mouse anti-IMC1 and rabbit anti-GAP45 were maintained in our laboratory.

## Results

### Binding ability prediction of TgGST2 to exogenous progesterone and estradiol

Mammalian GST is involved in steroid synthesis and metabolism, such as catalyzing the metabolism of Δ^5^ precursors to progesterone and dehydroepiandrosterone (DHEA) [9]. As such, we wanted to know whether TgGST2 could combine with hormones and function in the form of vesicles or granules to regulate protein transport and thus affect the survival of parasites. As a result, we first predicted the binding ability of TgGST2 to P4 and E2 via bioinformatics methods.

**Fig. 1:**
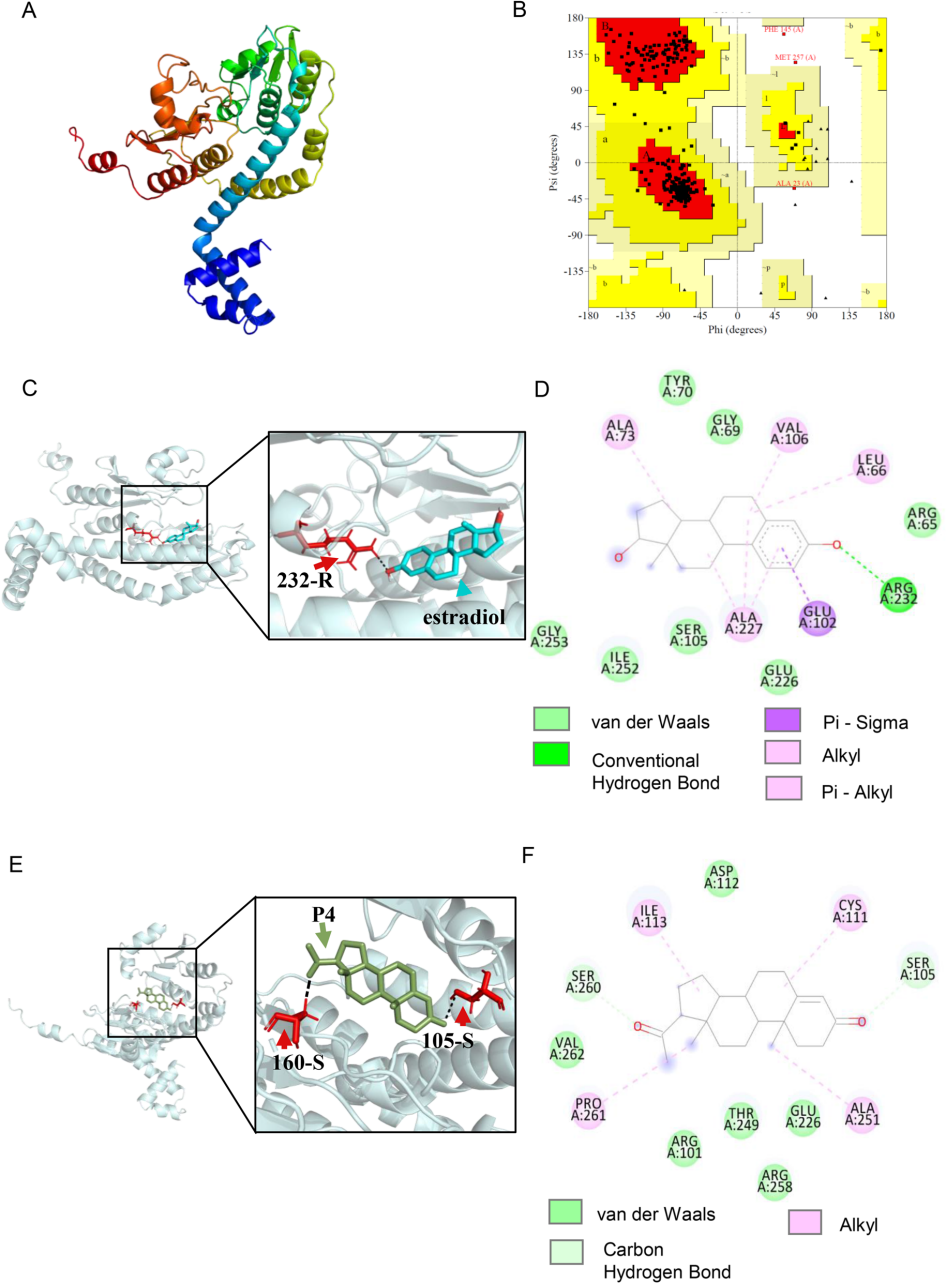
Binding ability prediction of TgGST2 to exogenous progesterone and estradiol. **A**–**B** The 3D structure predication of TgGST2. **C**–**D** Molecular docking of TgGST2 and estradiol with positive binding ability. Hydrogen bonds play a major role between the hydroxyl terminal of the benzene ring of E2 and the arginine of the amino acid at position 232 of TgGST2. **E**–**F** Molecular docking of TgGST2 and progesterone with relatively weak binding ability; the hydroxyl groups on both sides of progesterone interact with serine 105 and serine 160 of TgGST2, respectively

The 3D structure of TgGST2 was predicted via trRosetta with a score of 0.462. The quality of the model was evaluated by the Ramachandran plot, and 93% of the residues were in the appropriate regions (Fig. 1A, B). This indicates that the established tertiary structure model of TgGST2 is of good quality. AutoDock Vina docking predicted a positive binding ability of TgGST2 to estradiol of about –7.9 kcal/mol. Hydrogen bonds play a major role between the hydroxyl terminal of the E2 benzene ring and the arginine of the TgGST2 amino acid at position 232 (Fig. 1C, D). The binding energy of TgGST2 to P4 was –7.8 kcal/mol. The hydroxyl groups on both sides of progesterone interacted with Ser 105 and Ser 160 of TgGST2, respectively, but the interaction was slightly weaker (Fig. 1E, F).

### Influence of E2 and P4 on TgGST2

**Fig. 2:**
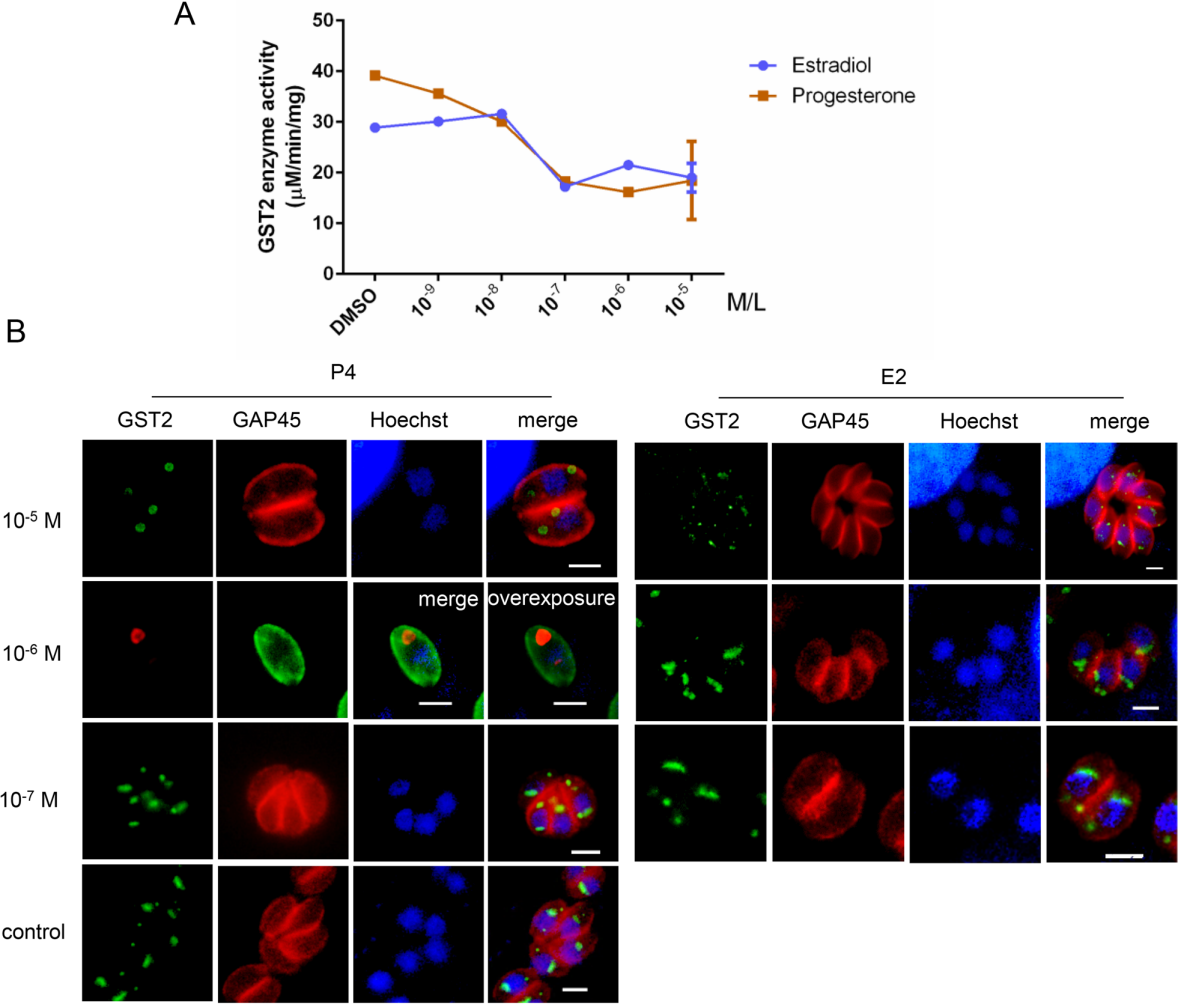
The influence of progesterone and estradiol on the enzyme activity and localization of TgGST2. **A** The significant decrease in enzymatic activity of rTgGST2 after progesterone and estradiol treatment, and the inhibitory effect is enhanced with increasing hormone concentration. **B** Localization of TgGST2 treated with P4 and E2, using DMSO as control. Under 10^–5^ M P4 treatment, the Golgi-located TgGST2 disappeared and vesicle-TgGST2 became vacuolated; under overexposure to 10^–6^ M of P4, vesicle-TgGST2 was vacuolated, Golgi-localized TgGST2 was only observed; otherwise, the same as the control group. HA (green), anti-mouse antibody; GAP45 (red), anti-rabbit antibody; Hoechst (blue); scale bar, 2 μm

To determine whether TgGST2 can respond to E2 and P4, the enzyme activity of recombinant TgGST2 protein (rTgGST2) was performed. Using DMSO as a control, rTgGST2 was incubated with different concentrations of estradiol and progesterone (10^–5^ M, 10^–6^ M, 10^–7^ M and 10^–8^ M) at 37 ℃ for 1 h, and then the absorbance values of GSH and CDNB catalysis were examined at 340 nM [22]. The results showed that P4 and E2 could inhibit the enzyme activity of rTgGST2, depending on their concentrations (Fig. 2A).

HFF cells were infected with endogenous HA-labeled TgGST2 parasites (TgGST2-HA) and cultured with DMEM containing 2% FBS and different concentrations (10^–5^ M, 10^–6^ M and 10^–7^ M) of hormones for 20 h. Indirect immunofluorescence assay (IFA) revealed that 10^–6^ M is the critical concentration at which P4 affects TgGST2, vesicle-localized TgGST2 becomes hollow rings, and the fluorescence intensity of Golgi-localized (short rod-like localized TgGST2 prior to the nuclei) TgGST2 is significantly weakened, a phenomenon that can only be observed under strong exposure (Fig. 2B). At 10^−5^ M, the Golgi-localized TgGST2 was completely eliminated, leaving hollow vesicles irregularly distributed in the cytoplasm. When P4 was 10^–7^ M, the localization of TgGST2 was the same as that of the DMSO control group in the form of both Golgi localization and solid vesicle localization (Fig. 2B). However, all concentrations of E2 had no effect on TgGST2, which is consistent with the control group (Fig. 2B). The above results suggest that TgGST2 responds to the treatment with progesterone and may act in the form of vesicles. This provides us with clues about how progesterone inhibits parasite life activity, possibly because of interference by TgGST2.

### Effect of progesterone on secretion-related proteins

**Fig. 3:**
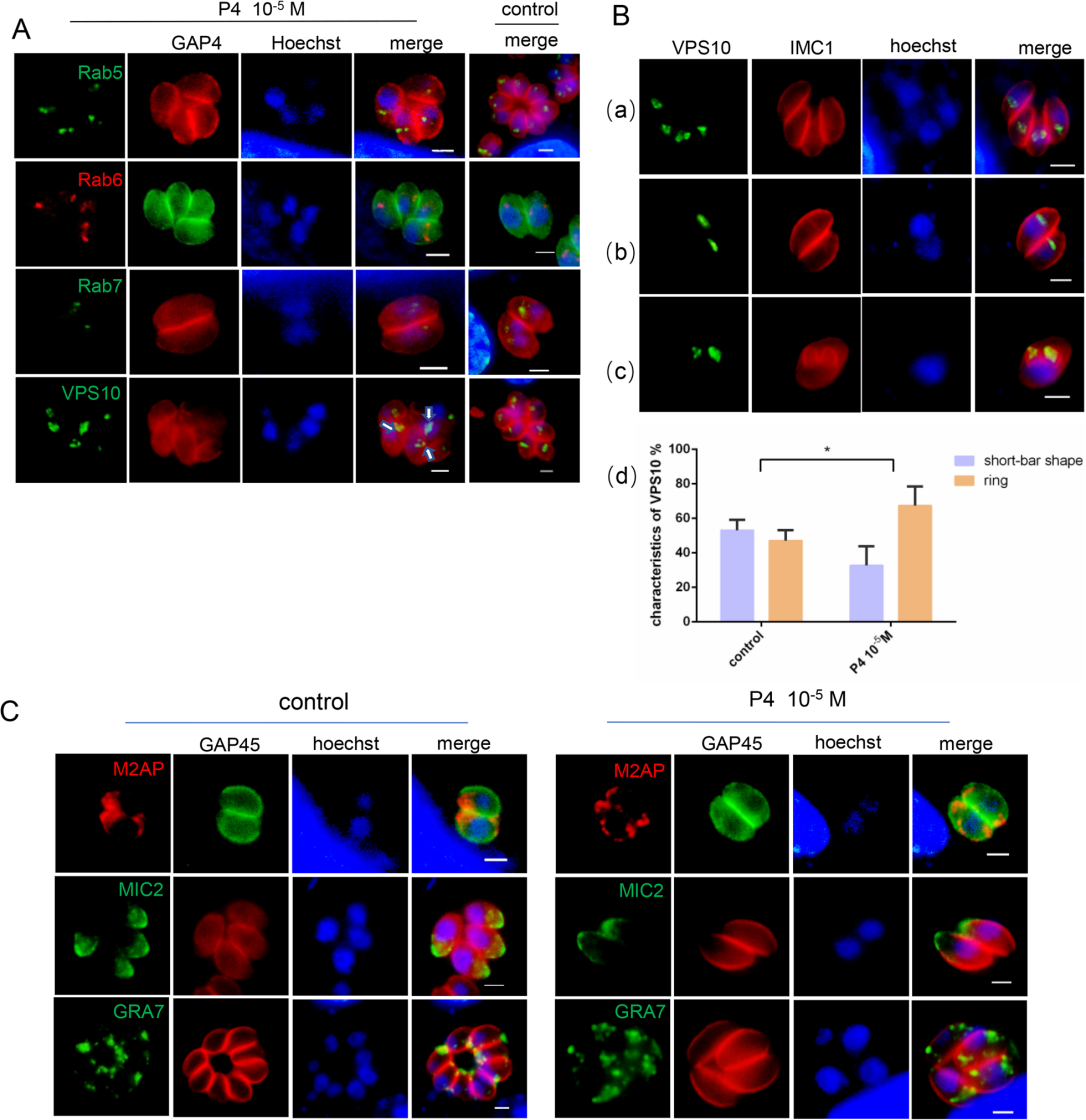
Progesterone causes abnormal localization of secretory protein transporter VPS10, microneme protein M2AP and MIC2. **A** Compared with the control group, the localization of Rab5, Rab6 and Rab7 was not affected by P4 treatment, while VPS10 displayed more ring localization (white arrow); target proteins were stained with anti-mouse FLAG antibody; GAP45, anti-rabbit antibody; Hoechst (blue); scale bar, 2 μm; **B** VPS10 showed two localization forms: **a** circular trajectories and **b** short-bar shape. **c** This localization was independent of whether the parasite was in a divided state or not; **d** the proportion of ring trajectory localization of VPS10 under P4 was significantly increased; VPS10, anti-mouse FLAG antibody; IMC1, anti-rabbit antibody; **D** P4 alters the localization of M2AP, which is continuous in the cytoplasm near the outline of the parasite, and MIC2 is more likely to accumulate on the parietal membrane of the parasite, while GRA7 is unaffected; FLAG, anti-mouse antibody, GAP45, anti-rabbit antibody; Hoechst (blue); scale bar, 2 μm

Since the localization of TgGST2 with P4 at 10^–5^ M concentration is similar to that of the protein transport inhibitor Brefeldin A (BFA) treatment by becoming hollow circles [20], we hypothesized that the inhibition of TgGST2 by progesterone may have the same mechanism as BFA, which affects protein transport by disrupting the Golgi apparatus. Thus, using proteins affected by BFA (Rab5, Rab6, Rab7 and VPS10) as controls, we found that Rab5, Rab6 and Rab7 are not affected by P4, but most of VPS10 proved to be circular or trajectory localized (Fig. 3A).

VPS10 is a pivotal protein in the secretory protein traffic or organelle biogenesis pathway, which is responsible for transporting immature secretory vesicles to different destinations for further processing and maturation. It binds to rhoptry or microneme proteins in the Golgi lumen and guides them through the endosome system to releasing them. The "empty" cargo receptor VPS10 then recruits components of the retromer complex for retrograde translocation and recycling to the Golgi to reload with new cargo [23], making VPS10 form a circular track-like localization in the parasite. During our observation of P4-treated VPS10, we found that it has two forms: ring trajectory (Fig. 3B a) and short-bar type (Fig. 3B b). The two types' localization of VPS10 was independent of whether the parasite was in a divided state or not (Fig. 3B c); both were found in the control and experimental groups, and statistical analysis showed that the number of ring trajectory localizations under P4 treatment was significantly higher than in the DMSO group (Fig. 3B d). Moreover, the MIC2-associated protein (M2AP) changed from its typical microneme location to continuous dots distributed along the internal contour of the parasite, while MIC2 seemed to be more easily concentrated on the apical membrane of the parasite (Fig. 3C). This suggests that the secretory protein trafficking is inhibited by P4, which may be an important reason for the reduced motor ability of the parasites.

### TgGST2 response to variations in different drugs

Eukaryotic GSTs can catalyze the combination of GSH with toxic compounds and detoxify them through the removal of transmembrane-related proteins such as multidrug resistance-associated protein 1(MRP1) or MRP2 from the cell [24]. To determine whether TgGST2 has a similar function, we observed its localization with different types of drugs (the drugs used are shown in the Table 1). These drugs were divided into the following categories: calcium ion antagonist nifedipine (pathogen-independent drugs); antimycotic (voriconazole and clarithromycin); antiviral (AIDs) including ritonavir, darunavir and indinavir; anticarcinogen (KU-19, Amg-319, WYE-132 and SH5-07); coccidiostats (dinitolmide, diclazuril, toltrazuril and metronidazole). The choice of these drugs is largely based on laboratory preservation.

**Table 1:** Compound information

Classify	Name	Mechanism	Function and application
Calcium ion antagonist	Nifedipine	Inhibits the activity of phosphodiesterase	Coronary heart disease, angina pectoris and severe hypertension
Antifungal agents ^a^	Voriconazole^a^, clarithromycin ^a^	Inhibits protein synthesis	A broad-spectrum antifungal
Antiviral agent	Ritonavir, darunavir, indinavir	Inhibitors of aspartate protease	AIDs
Anticarcinogen ^a^	① KU-60019^a^, ② Amg-319^a^, ③ WYE-132^a^, ④ SH5-07^a^	Undefined	An effective radiosensitizer helps to kill cancer cells
Coccidiostat	Dinitolmide^a^, diclazuril^a^, toltrazuril, metronidazole	Undefined	Have special effect on prevention and treatment of coccidiosis

^a^Drugs which could inhibit parasite growth

**Fig. 4:**
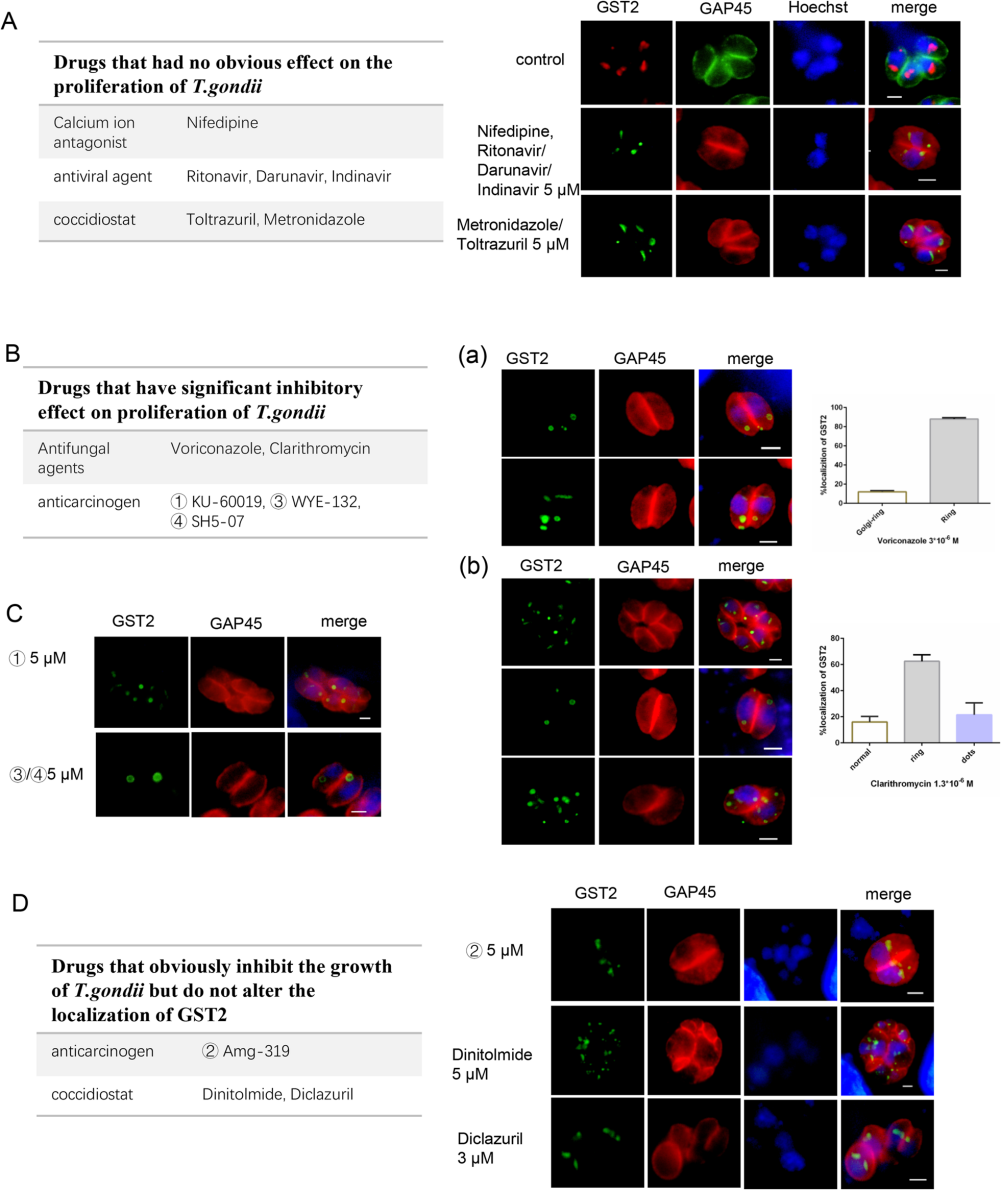
Effects of different drugs on TgGST2. **A** Drugs having no significant effect on the proliferation of *T. gondii* include nifedipine, antiviral agents and coccidiostats toltrazuril and metronidazole (5 μM), which also have no influence on the localization of TgGST2 (Golgi and dots); **B**–**C** Antifungal agents (voriconazole, clarithromycin) and some of the anticarcinogens (① KU-19, ③ WYE-132, ④ SH5-07) can significantly inhibit the growth of parasites causing unusual localization of TgGST2. **C** Some drugs (anticarcinogen ② Amg-319, coccidiostats dinitolmide and diclazuril) can affect the proliferation of parasites but have no influence on the localization of TgGST2; HA (green), anti-mouse antibody; GAP45 (red), anti-rabbit antibody; Hoechst (blue); scale bar, 2 μm

Their working concentrations against parasites were explored via plaque assays to estimate whether they would affect the growth of parasites. Nifedipine (5 μM) and antiviral agents (5 μM) had no effect on the parasite and were not significantly different from the control; the antifungal agents voriconazole (1.3 μM) and clarithromycin (3 μM) had an obvious effect on the survival of the parasites; anticarcinogen ① KU-19 (2 μM), ③ WYE-132 (5 μM) and ④ SH5-07 (5 μM) all inhibited the growth of the parasites, while ②Amg-319 (5 μM) showed no significant difference; the coccidiostats metronidazole (5 μM) and toltrazuril (5 μM) showed no significant difference from the control group, but dinitolmide (10 μM) and diclazuril (3 μM) obviously affected the survival of the parasites (Additional file 1: Figure S1). Based on the influence of these compounds on *Toxoplasma*, we divided them into drugs with inhibitory effects on the proliferation of parasites (drugs with the “a” label) and drugs with no significant effect. Among them, nifedipine, antiviral agents and the coccidiostats toltrazuril and metronidazole had no obvious effect on the parasites or on TgGST2, which possessed both Golgi- and vesicle-localized forms just like the control (Fig. 4A).

In contrast, for drugs that could inhibit the proliferation of *Toxoplasma*, such as antimycotics, anticarcinogens and coccidiostats (dinitolmide and diclazuril), they could also lead to disordered localization of TgGST2. TgGST2-HA parasites were cultured in the presence of the antibiotic voriconazole (3 × 10^–6^ M) for 20 h. TgGST2 exhibited two forms of localization that differed from the control, with statistical analysis showing the proportion of Golgi + hollow- ring (Golgi-localized and hollow ring-like localized TgGST2) to be ~ 12% and the complete hollow ring-like localized TgGST2 to be almost 88% (Fig. 4B a). The effect of clarithromycin was somewhat different from that of voriconazole, with TgGST2 showing three states at 3 × 10^–6^ M concentration, having multi-dot localization accounting for about 21.5%, the complete hollow ring being ~ 62.5% and ~ 16% being normal (Fig. 4B b). With the treatment of anticarcinogen ①KU-60019, TgGST2 exhibited two forms of localization (Golgi and hollow circle localized), while ③ WYE-132 and ④ SHE5-07 caused the disappearance of Golgi-localized TgGST2 and the dots were disseminated in the cytoplasm as a hollow ring (Fig. 4C). Based on the above research, we can confirm that TgGST2 can process different exogenous hormones or drugs, which varies with the characteristics of the substance. However, not all drugs that influence the proliferation of parasites can alter the localization of TgGST2. Although the use of the coccidiostats dinitolmide (5 μM) and diclazuril (3 μM) causes aberrant division of parasites, the localization of TgGST2 remains unchanged and is expressed in two types: Golgi and vesicles. Moreover, the anticarcinogen Amg-319 also did not affect TgGST2 (Fig. 4D). In general, our data suggest that TgGST2 is selective in its ability to respond to or process different drugs. However, the exact function of these vesicles produced by drug stress is still not fully understood by us and needs to be studied with more effort.

## Discussion

Previous studies on *Toxoplasma* GST have been limited to the immunogenicity of recombinant proteins expressed in *E. coli*, whose function and mechanism have not been reported [25]. Our initial research revealed that *T. gondii* contains three GST proteins, of which TgGST2 plays an important role in parasite proliferation. It is located in the Golgi-endosomal system and vesicles in the form of a membrane protein involved in the secreted proteins trafficking. Knocking out TgGST2 leads to the abnormal localization of part of the vesicle transport-related factors and some secreted proteins. In addition, TgGST2-deficient parasites were significantly less invasive and pathogenic in mice [20].

Various hormones working in a protein-bound form, such as prolactin (PRL) or sex hormone-binding globulin (SHGB), are also known as protein hormones. They are stored in large protein-filled vesicles, often called secretory granules. The granules are retained until they are stimulated and then the contents are released by exocytosis so that the hormones are rapidly available when needed [21]. Mammalian GSTs have been shown to be involved in steroidogenic metabolism through co-interactions with other proteins. Steroidogenic factor 1 (SF-1) binds directly to the human GSTA3 and GSTA4 promoters, together with 3β-HSD II, and catalyzes the metabolism of Δ^5^ precursors to progesterone and DHEA [26]. Plant GST is also a carrier and binding protein of auxin indoleacetic acid (IAA), a non-enzymatic interaction that temporarily stores and regulates the activity of IAA or helps its transport from the membrane to the receptor to regulate physiological activity [27]. During the parasitic disease schistosomiasis, sex hormones have an important influence on the age- and gender-dependent levels of infection. Lille et al. demonstrated that the 28-kDa GST of *Schistosoma haematobium* has high affinity binding to testosterone, which has the ability to inhibit its enzymatic activity in a dose-dependent manner, leading to a reduction in *Schistosoma* fecundity, suggesting that this hormone may be directly involved in an anti-fecundity mechanism [28].

*Toxoplasma* is more susceptible to pregnant women and causes reproductive disorders. Therefore, changes in hormone levels may be a critical element of high infectivity and pathogenicity. Our team’s previous investigation on the influence and mechanism of hormones on *Toxoplasma* showed that progesterone could significantly reduce the secretion of MIC2 and inhibit its gliding ability. As such, we wanted to determine whether TgGST2 could bind to hormones and in turn affect the life activities of the parasite. With this in mind, we used a molecular docking method to predict the binding ability of TgGST2 to estradiol and progesterone, respectively. This indicates that TgGST2 has better binding ability to estradiol and weaker binding ability to progesterone. Actually, these predicted results confused us to some extent, because P4 showed stronger inhibition regardless of the enzymatic activity of rTgGST2 protein and on the protein itself, which is still not fully understood. In any case, we can confirm that TgGST2 has the potential to bind distinctively to hormones. The dose-dependent inhibition of rTgGST2 enzyme activity by the hormone suggests that they are indeed correlated.

To verify the above findings, we observed the changes in TgGST2 after two different hormone treatments through IFA approach. We found that the localization of TgGST2 changed significantly at 10^−6^ M and 10^−5^ M of progesterone, somewhat similar to the gradual disappearance of Golgi-localized GST2 with increasing P4 concentration, which indicated that TgGST2 responded to exogenous P4, but there was no obvious change in TgGST2 after treatment with E2, probably due to different metabolic pathways and mechanisms. Since the effect of 10^–5^ M P4 on GST2 is consistent with the treatment of BFA, we wondered whether their mechanisms are congruent. However, the results differed from our prediction, as the proteins affected by BFA did not seem to change significantly when treated with P4 except for an increase in the proportion of VPS10 circular trajectories. VPS10 is a critical transporter in secretory organelles or protein trafficking pathways, responsible for delivering different proteins to their respective destinations and then coming back to the Golgi lumen for a new “journey.” The increased circular trajectories imply that P4 may impede the transport rate of secreted proteins or, alternatively, that it facilitates the transport of substrates, which requires more investigation.

Exogenous hormones belong to a class of drugs. Since TgGST2 has the capability to deal with exogenous P4, what about other types of drugs? We selected four different types of drugs used in our laboratory to observe changes in TgGST2 or to detect the ability of TgGST2 to respond or process. We found that these drugs were not involved in parasite growth and did not influence TgGST2. However, when we increased the concentration of nifedipine, we could observe that vesicles of GST2 were transported outside the parasites (data not shown). It consists of GSTP1 in human cells that catalyzes the binding of GSH to xenobiotics and its excretion outside the body via the multi-drug resistance protein (MPR). Drugs that obstruct parasite proliferation may alter the localization of TgGST2; more importantly, these changes are divergent. However, not all drugs that induce division disorders lead to a transformed localization of TgGST2, and it is possible that TgGST2 prefers certain drug components, but this selectivity is currently unclear and may be related to the structure of both TgGST2 and chemicals. Do the inhibitory effects of drugs as well P4 on the parasite lead to changes in TgGST2 localization or does drug treatment disrupt TgGST2 thereby resulting in compromised parasite survivability? How were these hollow vesicles produced, and what role would they play? We still do not fully understand, and more studies are needed.

## Conclusions

Our study found that TgGST2 could bind to exogenous E2 and P4 and that enzymatic activity was inhibited by the hormones in a concentration-dependent manner. Upon P4 treatment, TgGST2 changed from Golgi and vesicles localized to hollow circles, leading to abnormal localization of VPS10, M2AP and MIC2, but E2 had no significant effect. Moreover, diverse types of drugs had divergent effects on TgGST2, among which treatment with antifungal agents (voriconazole and clarithromycin), anticarcinogens (KU-60019, WYE-132 and SH5-07) and coccidiostats (dinitolmide and diclazuril) made the localization of TgGST2 appear in different forms, including dots, circles and rods.

## Supplementary Information

**Additional file 1: Figure S1.** Treatment of *T. gondii* with different drugs; 350 tachyzoites of *Δku80* parasites were seeded into six-well plates covered with HFF cells, cultured with DMEM containing different drugs for 7 days, and then fixed and stained; the use of nifedipine at a concentration of 5 μM showed no significant difference compared with the control (DMSO/ ethyl alcohol); the antifungal agents voriconazole at 1.3 μM and clarithromycin at 3 μM both obviously affected the survival of the parasites; anticarcinogen ① KU-19, ③ WYE-132 (5 μM) and ④ SH5-07 (5 μM) all inhibited parasite growth, while ② Amg-319 (5 μM) showed no significant difference; coccidiostat metronidazole (5 μM) and toltrazuril (5 μM) did not differ significantly from the control, whereas dinitolmide (10 μM) and diclazuril (3 μM) obviously inhibited the survival of parasites.

## Data Availability

All datasets generated for this study are included in the manuscript/Additional files.

## Abbreviations

GST: Glutathione S-transferase
E2: Estradiol
P4: Progesterone
VPS10: Sortilin
MIC2: Microneme protein 2
M2AP: MIC2-associated protein
CDNB: 1-Chloro-2, 4-dinitrobenzene
IFA: Indirect immunofluorescence assay
BFA: Brefeldin A
Δ: Gene knockout
